# A Chemometric Exploration of Potential Chemical Markers and an Assessment of Associated Risks in Relation to the Botanical Source of Fruit Spirits

**DOI:** 10.3390/toxics12100720

**Published:** 2024-10-02

**Authors:** Branislava Srdjenović Čonić, Nebojša Kladar, Dejan Kusonić, Katarina Bijelić, Ljilja Torović

**Affiliations:** 1 Department of Pharmacy, Faculty of Medicine, University of Novi Sad, Hajduk Veljkova 3, 21000 Novi Sad, Serbia; branislava.srdjenovic-conic@mf.uns.ac.rs (B.S.Č.); nebojsa.kladar@mf.uns.ac.rs (N.K.); dejan.kusonic@mf.uns.ac.rs (D.K.); ljilja.torovic@mf.uns.ac.rs (Lj.T.); 2 Center for Medical and Pharmaceutical Investigations and Quality Control, Faculty of Medicine, University of Novi Sad, Hajduk Veljkova 3, 21000 Novi Sad, Serbia

**Keywords:** stone fruits, pome fruits, grape pomace, spirit composition, chemometric analysis, margin of exposure

## Abstract

Chemometric evaluation of potentially harmful volatile compound and toxic metal(loid) distribution patterns in fruit spirits relating to distinct fruit classes most commonly used in spirit production highlighted the potential of several volatiles as candidates for differentiation markers while dismissing toxic metal(loid)s. Pome fruit and grape pomace spirits were mostly characterized by a lower abundance of n-propanol, methanol, ethyl acetate and acetaldehyde, while stone fruit spirits contained lower amounts of isoamyl alcohol and isobutanol. Chemometric analysis of the fruit spirit composition of aromatics identified additional potential markers characteristic for certain fruits—benzoic acid ethyl ester, benzyl alcohol, benzaldehyde, butanoic acid 3-methyl-ethyl ester, butanoic acid 2-methyl-ethyl ester and furfural. This study explored the variability in the risk potential of the investigated spirits, considering that some chemicals known to be detected in spirits are potent health hazards. Ethyl carbamate in combination with acetaldehyde showed a higher potential risk in stone fruit spirits, methanol in stone and pome fruit spirits and acetaldehyde in grape pomace spirits. It is of great interest to evaluate to what extent consumers’ preference for spirits of distinct fruit types affects health risks. Consumers of stone fruit spirits are potentially at higher risk than those consuming pome fruit or grape pomace spirits.

## 1. Introduction

Alcohol consumption in 2019 contributed to 2.6 million deaths globally, accounting for 4.7% of all deaths. Furthermore, among males, 2 million alcohol-attributable deaths and 6.9% of all disability-adjusted life years (DALYs) were reported, while in females these numbers were 0.6 million deaths and 2.0% of all DALYs [1]. 

According to the WHO data, alcohol consumption is the most widespread habit on the European continent, and 5.5% of all deaths were a result of excessive consumption of alcoholic beverages, while light to moderate alcohol consumption correlated with almost 23,000 new cancer cases in 2017 (nearly half of these were female breast cancers) [1,2,3]. The common opinion in the general population is that light alcohol consumption could have some beneficial effects; thus, alcohol consumption still represents a common habit and a socially acceptable behavior in the population of most European countries. Alcohol may decrease the risk of diabetes mellitus (in women only), ischemic heart disease and ischemic stroke in low-level alcohol consumers and those not engaged in heavy episodic drinking (as compared to lifetime abstainers) [1]. However, no studies have shown that any amount of alcohol could reduce the risk of cancer for an individual consumer. It is important to highlight that according to the most recent official position of health authorities, no amount of alcohol consumed is without health risk [4]. Worldwide, spirit consumption accounts for 45.4% of the total alcohol consumption, and it is proven to be the factor with the greatest impact on the death rate of adult men in Europe, especially when the consumption of unrecorded spirits is taken into account [1,5]. We are all witnessing the flood of alcoholic beverages on the market, and consumers rightly expect that they are safe, at least in terms of the presence of harmful chemicals other than ethanol. Ethanol remains their own choice—in this regard, mandatory labeling of the presence and amount of ethanol makes this choice an informed one, at least from the perspective of the producers. In addition to regulatory activities, health professionals and authorities can express their views through alcohol awareness campaigns and thus motivate the population to stop consuming alcohol.

The most important compounds besides ethanol that can be found in spirits and that possess potentially negative effects on human health are methanol, volatile higher alcohols, ethyl acetate, toxic metals and those with pronounced carcinogenic potentials—ethyl carbamate and acetaldehyde [6,7,8,9].

During fruit spirit production, ethanol is the main product of alcoholic fermentation. As a genotoxic carcinogen, it represents a toxic substance of the greatest health risk in spirits [10]. Methanol, originated from pectins, is a natural ingredient with a major occurrence in fruit spirits. Plum, apple and pear fruits contain a higher portion of pectins than grapes do [11]. Since 2012, methanol has been on the list of chemicals that cause developmental toxicity [12]. Higher alcohols are a group of aromatic and aliphatic alcohols formed as products of sugar and amino acid metabolism during alcoholic fermentation. They affect the aroma profile and are precursors in ester production. Of more than 40 higher alcohols, the most important are 1-propanol, 1-butanol, 2-butanol, isobutanol and isoamyl alcohol. It was proven that specific types of liquor from India that are rich in higher alcohols exhibited greater toxicity in rats than the equivalent dose of pure ethanol [13]. Higher alcohols were proposed to be a reason for unrecorded alcohol toxicity in Eastern Europe [14]. Ethyl acetate, formed as a result of sugar metabolism during fermentation, is the most important representative of the esters group in fruit spirits, which give it a pleasant, fruity–floral aroma. There is no evidence of its carcinogenicity, but ethyl acetate hydrolyzes to ethanol and acetic acid, contributing to the toxicity of ethanol in fruit spirits [9]. Acetaldehyde is a carbonyl compound formed during fermentation as an intermediate product of ethanol formation. In smaller quantities, it contributes to a pleasant fruity aroma, while in larger quantities, it gives an unpleasant taste [9,15]. Acetaldehyde, when occurring as a regular ingredient in a large number of foods, is classified as possibly carcinogenic to humans (group 2B), while as an alcoholic beverage ingredient, it is classified as a human carcinogen (group 1) [10]. Ethyl carbamate, formed from cyanogenic glycosides as the most important precursors predominantly found in stones of fruits [16,17], is classified as a probable human carcinogen (group 2A) [18].

The relevant requirements for fruit spirits, including “rakija” (a Serbian domestic diluted spirit), as stated in the Regulation of the Republic of Serbia and harmonized with EU Regulation 2019/787, are as follows: alcoholic strength by volume: 37.5 to 86%; volatile substance content: at least 200 g/hL of 100% vol. alcohol; methanol content: max. 1000, 1200 or 1350 g/hL of 100% vol. alcohol, depending on the fruit type used for production [19]. Additionally, Regulation of the European Council (EC) 110/2008 defines spirits as “a drink produced exclusively by the alcoholic fermentation and distillation of fleshy fruit or must of such fruit, berries or vegetables, with or without stones”. The alcohol content in given spirits shall range from 37.5 to 86% *v*/*v*, while their flavor must be derived from the fruit materials used in production [20]. Maximum chemical testing limits for substances with potentially harmful effects on human health in recorded and unrecorded alcoholic beverages proposed by the Alcohol Measures for Public Health Research Alliance project (AMPHORA) expressed as g/hL of 100% vol. alcohol are for methanol 1000, higher alcohols (in sum) 1000, ethyl acetate 1000 and acetaldehyde 50 [21].

Analysis of fruit spirits is critically important, not only to comply with legal requirements but also due to the plethora of compounds affecting the quality of the stated products [22]. Fruits primarily contribute to aromatic substances that differentiate one fruit spirit from another. Defining the compound characteristics of different fruit spirits could improve the quality control of this type of product since its production often represents a source of great economic profit, but it is also an area susceptible to manipulation, especially regarding unregistered production. It is not uncommon to blend high-quality distillates with cheaper raw materials of lower quality or to use low-quality production facilities. Thus, it is important to identify marker substances that can facilitate product quality assessment and manufacturing process monitoring. It is possible, using appropriate analytical and statistical methods, to distinguish spirits obtained from different fruits such as sweet cherry, sour cherry, pear, apple, apricot or plum [23].

Data collected over 3 years (2016–2018) showed that the number of alcohol-related deaths in Serbia was 2500 annually (a prevalence four times higher in men). Also, the highest rate of deaths was shown in northern Serbia (the Autonomous Province of Vojvodina) [24]. The Balkans as well as regions of Central and Southeast Europe are well-known for extensive production and consumption of fruit spirits produced in private homes or small-scale distilleries, which are not subjected to official quality control. The yearly production of these product types in Serbia is around 50 million liters [25]. This results in a highly diverse offering of such spirits, with the most common being produced from plums, grapes and pome fruits. In 2022, the traditional Serbian plum spirit “šljivovica” was added to the UNESCO Representative List of the Intangible Cultural Heritage of Humanity. Besides wide consumption during festive and family gatherings, šljivovica plays a significant role in traditional medicine, where medicinal herbs or fruits are incorporated into cold and pain remedies or antiseptics [26]. 

Comprehensive analysis regarding harmful volatiles and toxic elements in fruit spirits originating from Serbia by our investigation group was focused on differences between recorded and unrecorded rakija [7,8]. However, a study of ethyl carbamate that explored the influence of different fruit classes on ethyl carbamate content in fruit spirits and consequent health risks indicated the necessity of such analysis with regard to other hazardous substances detected in rakija [27]. Additionally, considering that many people prefer drinking rakija produced from a specific fruit (mostly apricot, quince or plum), it is justified to explore whether such preferences cause a higher risk for some of them.

The aims of the current study were as follows: (1) exploration of the patterns of distribution of potentially harmful volatile compounds and toxic elements relating to selected fruit classes (pome fruits, stone fruits, grape pomace) used for spirit production by application of chemometric analysis; (2) assessment of risk related to fruit spirits associated with hazardous substances having different patterns of distribution in investigated fruit classes in terms of non-compliance with limit levels recommended by AMPHORA and health risk caused by fruit spirit consumption; (3) compositional analysis of aromatic compounds in spirits obtained from specific fruits; and (4) identification of compounds having the potential to be applied as quality markers for differentiation of specific fruits used for spirit production by chemometric analysis.

## 2. Materials and Methods

### 2.1. Fruit Spirit Samples Information

The research included 106 samples of the fruit spirit “rakija” (Serbian traditional alcoholic drink) collected in the period 2020–2022 in the territory of the Serbian Autonomous Province of Vojvodina. The samples were collected in well-closed glass bottles and kept in the dark at room temperature until the analysis. Collected samples included recorded spirits (commercially available, made in Serbia) and unrecorded spirits (obtained directly from producers from their home production facilities and small-scale distilleries), produced from various fruit sources which were classified into three fruit classes: group G1, pome fruits (apple, pear, quince; n = 24); group G2, stone fruits (plum, peach, apricot, cherry; n = 62); group G3, grape pomace (n = 20).

### 2.2. Utilization of Previously Published Data

The current study was partly based on previously published data obtained from the same set of spirit samples. The contents of ethanol, methanol, acetaldehyde, ethyl acetate and higher volatile alcohols were taken from the study published by Srdjenović-Čonić, Kladar, Božin and Torović [7]; ethyl carbamate content from the study performed by Kusonić, Bijelić, Kladar, Torović and Srđenović Čonić [27]; and the elemental profile from the study published by Torović, Čonić, Kladar, Lukić and Bijelović [8].

### 2.3. Semiquantitative Analysis of Aromatic Compounds in Fruit Spirits

Semiquantitative aromatic compound analysis in collected samples involved the previously described method [28] based on gas chromatography coupled with mass spectrometry (HSS-GC-MS), with some modifications (Supplementary Material, Table S1.) The identification of compounds of interest was conducted by comparing the mass spectra to those from the NIST library, while their abundance was calculated in relation to the sum of all integrated peak areas. Only the compounds present in amounts ≥5% were used as input variables for further chemometric analysis. 

### 2.4. Chemometric Analysis

The obtained results were summarized in the form of a matrix with dimensions 124 × 69, which was used as input for several multivariate statistical techniques. The specified matrix contained results of semiquantitative analysis of spirit samples experimentally obtained in the present study (Section 2.3.), as well as results of sample characterization in terms of ethanol, methanol, acetaldehyde, ethyl acetate and higher volatile alcohols; ethyl carbamate; and metal and metalloids content from previously published studies (Section 2.2.). The applied multivariate statistical techniques included principal component analysis (PCA), canonical discriminant analysis (CDA) and cluster analysis utilized to display the patterns of initial samples’ variability by reducing the number of dimensions used for description of the initial dataset. Briefly, the application of the mentioned techniques results in the calculation of principal components or discriminant axes (depending on the applied analysis type) which correlate to initial variables and preserve a certain portion of initial dataset variability. However, each subsequently calculated dimension explains a lower proportion of variance when compared to its predecessor, thus suggesting that the first calculated dimension is loaded by the highest variance proportion and that the first two or three dimensions are usually suitable for the successful interpretation of results. Regarding the diversity and specific requirements of the applied techniques, some modifications of the initial dataset had to be applied before analysis, which are described in detail in the corresponding manuscript sections. 

### 2.5. Compliance Evaluation and Risk Assessment

Required data were retrieved from previously published studies (Section 2.2.). Hazardous substance content compliance was assessed against the limits proposed by AMPHORA [21]. An ANOVA test with post hoc Tukey’s HSD test and Chi-square test were used to compare the content of potentially toxic compounds depending on the fruit class used for the production.

A margin of exposure (MOE) approach was used to estimate the risk associated with hazardous substances via selected fruit class. The calculated MOE values were compared with the limits set by the European Food Safety Agency [29].

## 3. Results and Discussion

### 3.1. Exploration of the Patterns of Distribution of Hazardous Substances in Relation to Fruit Classes

**Figure 1:**
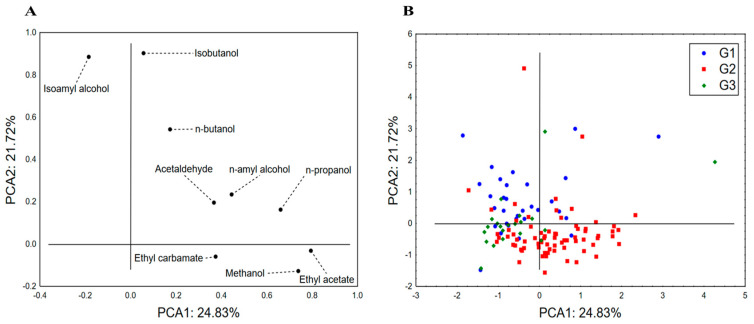
Principal component analysis—potentially harmful volatile substances in spirits: principal component loadings (**A**) and the position of analyzed spirits in the space defined by PCA1 and PCA2 (**B**). G1—pome fruits; G2—stone fruits; G3—grape pomace.

Different chemometric techniques were applied to the results of the chemical characterization of the analyzed spirits to identify the compounds highly correlating to the variability of the analyzed dataset and thus being the most responsible for differences between spirits obtained from selected fruit classes. The PCA applied to the dataset describing the content of potentially harmful volatile substances in spirit samples by taking into account their classification into groups G1–G3 shows that the first two principal components (PCA1 and PCA2) explain more than 45% of the original dataset variability (Figure 1). The recorded variability mostly correlates, in terms of PCA1, with the content of methanol, ethyl acetate and n-propanol, whereas the shape of the variability (in terms of PCA2) is a consequence of the isoamyl alcohol and isobutanol abundance. Samples’ positioning in the space defined by PCA1 and PCA2 (Figure 1) indicated no clear separative grouping of the spirits, but some trends in patterns of the analyzed chemical composition variability could be noticed. Namely, G1 and G3 samples are mostly located in the negative part of PCA1 as a result of the lower abundance of n-propanol, methanol, ethyl acetate and even acetaldehyde, while at the same time, G2 samples indicated high variability regarding the content of the previously mentioned compounds. Furthermore, in relation to PCA2, it can be noticed that G2 samples are mostly characterized by a lower abundance of isoamyl alcohol and isobutanol in relation to G1 samples, whereas G3 samples are, in terms of these alcohol contents, a “transitional group” between G1 and G3. Moreover, it can be noticed that although loadings of PCA1 and PCA2 by ethyl carbamate appear moderate, this compound additionally affects the positioning of stone fruit spirits (G2) in the positive part of PCA1 and the negative part of PCA2.

**Figure 2:**
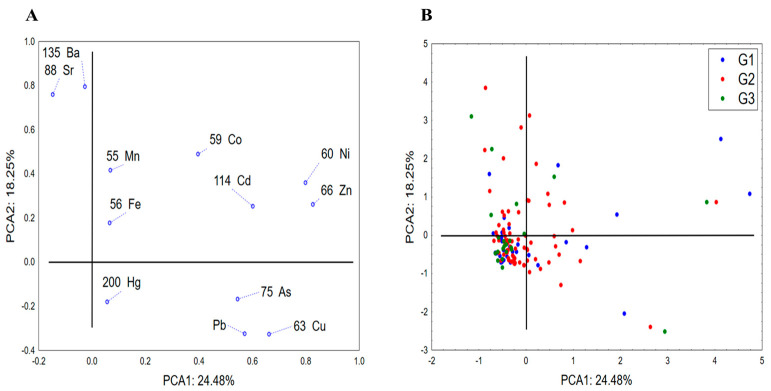
Principal component analysis—elemental profiling of fruit spirits: principal component loadings (**A**) and the position of analyzed spirits in space defined by PCA1 and PCA2 (**B**). G1—pome fruits; G2—stone fruits; G3—grape pomace.

The PCA application to the results of elemental profiling of analyzed fruit spirits showed that the first two PCAs describe around 43% of dataset variability, whereas the variability in term of PCA1 highly correlates to the concentration of Ni, Zn, Cu, Pb and Cd, while the shape of the variability is determined by amounts of Ba and Sr (Figure 2). The positioning of analyzed spirits in the space determined by PCA1 and PCA2 suggests no clear grouping related to specific patterns of accumulation of the stated elements in groups G1–G3, which indicates that the spirits’ elemental profiles are probably a function of the applied technological process for spirits production and are not related to the fruit raw material used. On the other hand, an encouraging fact could be that only a minority of the analyzed samples showed a high abundance of the stated metals and metalloids. 

### 3.2. Evaluation of Occurrence and Compliance Assessment of Hazardous Substances in Relation to Fruit Classes 

Chemometric analysis of the exploration of the patterns of distribution of hazardous substances in the selected fruit classes revealed that the spirits’ elemental profiles are probably a function of the applied technological process for spirits production and are not related to fruit raw material used, unlike potentially harmful volatile substances whose pattern of distribution highly depends on the fruit class. Thus, an assessment of risk related to the investigated classes of fruits was conducted only for potentially harmful volatile substances (ethanol, acetaldehyde, methanol, higher alcohols and ethyl acetate).

**Figure 3:**
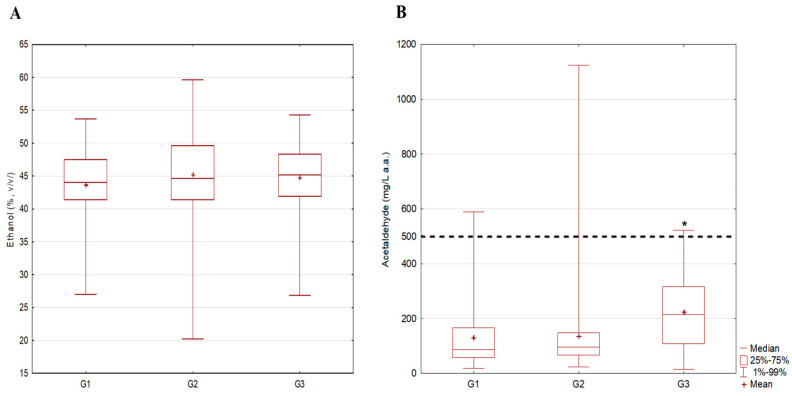
Comparison of measured content of (**A**) ethanol and (**B**) acetaldehyde in fruit spirits; * *p* < 0.05; ------------ AMFORA limit; G1—pome fruits; G2—stone fruits; G3—grape pomace.

**Figure 4:**
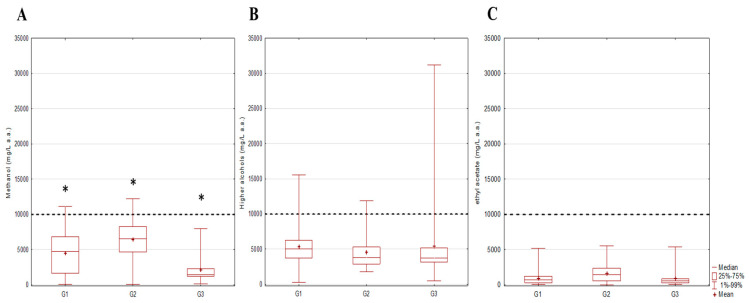
Comparison of measured content of (**A**) methanol, (**B**) higher alcohols and (**C**) ethyl acetate in fruit spirits; * *p* < 0.05; ------------ AMFORA limit; G1—pome fruits; G2—stone fruits; G3—grape pomace.

The analytically determined content of potentially harmful substances (except ethanol) is expressed in mg/L of absolute alcohol (a.a.) in order to compare concentrations in different alcohol strength samples. Contents of the carcinogenic compounds ethanol (%, *v*/*v*) and acetaldehyde are presented in Figure 3A,B, while contents of non-carcinogenic compounds (methanol, sum of higher alcohols and ethyl acetate) are presented in Figure 4A–C. 

The ethanol content in the analyzed samples ranged from 20 to 60% *v*/*v* and met the maximum limit of 86% *v*/*v* defined in Regulation 2019/787 of the EU and Regulation 110/2008 of the EC [19,20], while no statistically significant differences were observed between investigated spirits in relation to the fruit classes used for production (Figure 3A). The highest range was recorded within the G2 group, where plums were the dominant fruit in the production. The obtained results are similar to those presented by Jung, et al. [30], where the ethanol content in stone fruit spirit samples made from plum ranged from 52 to 76% *v*/*v*. Statistically higher content of acetaldehyde in group G3 vs. G2 (*p* = 0.034) (Figure 3B) was observed. In groups G1 and G3, only one sample had acetaldehyde content above the maximum limit of 50 g/hL (500 mg/L a.a.) proposed by AMPHORA, while in the G2 group, the number of such samples was two, and of these, the content of acetaldehyde in one of the samples was more than twice the limit (1124 mg/L a.a.). As in our study, a higher content of acetaldehyde in grape brandies in comparison to plum brandies was observed in the Bulgarian study of Dimitrov and Ivanova [31]. As was stated in our previous study, the highest range of ethyl carbamate was recorded in the G2 group—stone fruit spirit samples (0.005–15.36 mg/L a.a.)—where statistically higher content was observed in “rakija” produced from stone fruits in comparison to those produced from pome fruits (G1 group) (*p* = 0.041). The given results align with the results of a German study conducted on 631 stone fruit spirit samples. Namely, Lachenmeier, et al. [32] showed that the concentration of ethyl carbamate in given samples ranged from 0.01 mg/L to 18 mg/L, with a mean of 1.4 mg/L. The reason for the highest content of ethyl carbamate in spirits containing stone fruits could be the high residual cyanate levels in their seeds. Namely, the enzymatic and thermal cleavage of cyanogenic glycosides form cyanate, further reacting with ethanol and forming ethyl carbamate [16].

Regarding non-carcinogenic compounds, statistically significant differences in the methanol content were observed between all studied groups (G1 vs. G2 (*p* = 0.0007), G1 vs. G3 (*p* = 0.002) and G2 vs. G3 (0.00002)) (Figure 4A). The broadest range of methanol concentrations (20 mg/L to 12,192 mg/L a.a) with the highest mean value (6455 mg/L a.a.) was recorded in the G2 group. By analyzing 47 samples collected from the territories of five former member states of Yugoslavia, Mrvčić et al. [11] determined a maximum methanol content of 12,713 mg/L a.a. and stated the existence of a statistically significant difference between the samples produced from different types of fruit [11]. The lowest methanol content was recorded in grape pomace samples, which is in accordance with our findings that the lowest mean value of methanol content was in the G3 group—grape pomace spirit samples (2126 mg/L a.a.)—while the highest mean values were observed in the G2 group (6455 mg/L a.a.). Considering the raw materials used for fermentation, fruits are related to the highest methanol content in the spirit product due to their high pectin presence. Generally, fruit spirits produced from stone fruits of the genus *Prunus* (cherries, plums) and pome fruits of the genera *Malus* and *Pyrus* (apples and pears, respectively) have been correlated with the highest methanol content [33]. Regarding meeting the maximum methanol content of 10,000 mg/L [4,21], it was shown that one sample from the G1 group (11,097 mg/L a.a.) and two samples from the G2 group (11,280 and 12,192 mg/L a.a.) had a methanol content above the proposed limit. Winterová, Mikulikova, Mazáč and Havelec [23] reported similar results, where out of 153 analyzed samples, 8 stone fruit brandies and 21 pome fruit brandies exceeded the maximum methanol content. 

**Figure 5:**
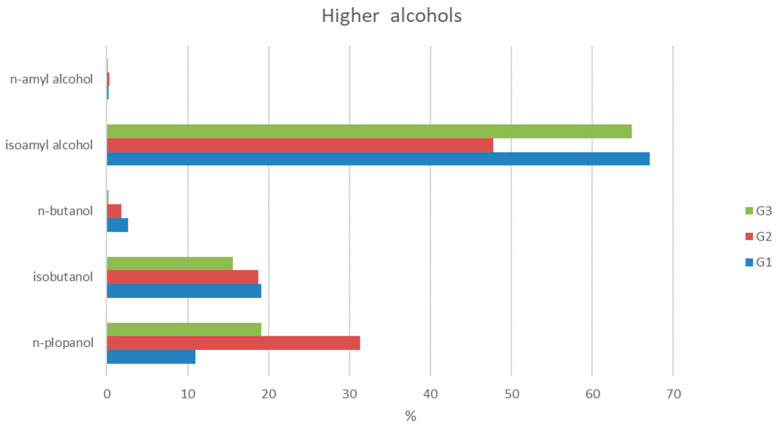
Average content (%) of individual higher alcohols in the total average content of higher alcohols in fruit spirits. G1—pome fruits; G2—stone fruits; G3—grape pomace.

**Figure 6:**
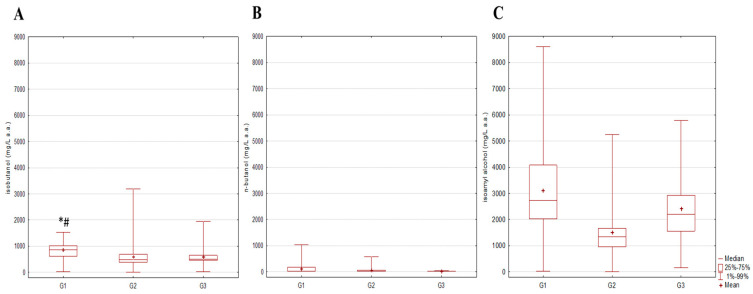
Measured content of individual higher alcohols in fruit spirits; (**A**)—isobutanol, (**B**)—n-butanol, (**C**)—isoamyl alcohol; *# *p* < 0.05; G1—pome fruits; G2—stone fruits; G3—grape pomace.

The highest concentration range of higher alcohols was recorded in the G3 group, with the highest value of 32,236 mg/L a.a (Figure 4B). The lowest mean value of the given higher alcohol content was in the G2 group (4546 mg/L a.a.). Although the mean values of the other two groups were higher (G1 at 5338 mg/L a.a. and G3 at 5455 mg/L a.a.), no statistical significance was observed between groups. These findings are in accordance with the higher alcohol content published by the previously mentioned group of authors, where the same trend of mean values was reported—lowest in the stone fruit spirit group and higher in grape spirit and groups of pome fruits (apple or quince spirit), without statistical significance among the observed groups [11]. In both studies, the most dominant higher alcohol in all tested fruit spirit categories was isoamyl alcohol (Figure 5), of which the lower percentage was observed in stone fruit spirits, 48%, compared to 67% and 65% in the G1 and G3 groups, respectively. In spirits obtained from pome fruits (G1 group), the lower quartile value was already 3719 mg/L a.a., and a high content of higher alcohols can influence the final product’s taste, potentially causing an unpleasant aroma, especially at concentrations above 3500 mg/L a.a. [34]. Four sample spirits from group G2 and two from each of the groups G1 and G3 exceeded the maximum concentration limit set by AMPHORA of 10,000 mg/L. As for the individual higher alcohol representatives, statistically higher content of isobutanol, n-butanol and isoamyl alcohol was observed in spirits produced from pome fruits in comparison to those obtained from stone fruits or grape pomace (isobutanol: G1 vs. G2 (*p* = 0.003) and G1 vs. G3 (*p* = 0.033); n-butanol: G1 vs. G2 (*p* = 0.029) and G1 vs. G3 (*p* = 0.007); isoamyl alcohol: G1 vs. G2 (*p* = 0.00002) and G1 vs. G3 (*p* = 0.003)) (Figure 6). Results from the investigation of Šekerić, et al. [35] revealed that better sensory quality of apple spirits (pome fruit spirits) correlated to higher alcohol concentration, while there was no difference for the plum or grape spirits. Thus, the producers often dilute fruit spirits with water to increase sales value by stretching production volumes, especially in the case of plum or grape spirits, since this action does not change their sensory characteristics [35]. Additionally, high levels of 1-butanol, 2-butanol, amyl alcohol and isoamyl alcohol generated a strong, pungent odor in spirits produced from the “Požegača” variety (type of plum cultivar) [36]. Regarding ethyl acetate content, the obtained results are in accordance with those published by Mrvčić et al. reporting that samples of all selected fruit classes contain ethyl acetate in amounts below the maximum allowed limit (Figure 4C) [11]. However, it is interesting that statistically higher content of ethyl acetate was observed in spirits produced from stone fruits in comparison to those obtained from pome fruits or grape pomace (G2 vs. G1 (*p* = 0.011) and G2 vs. G3 (*p* = 0.022)). Although no statistically significant difference between categories was observed in the study of Mrvčić et al. [11], stone fruit spirits had by far the highest mean value and range of spirit total esters compared to grape spirits or those made from pome fruits.

### 3.3. Risk Assessment of Hazardous Substances in Relation to Fruit Classes 

The present study aimed to evaluate whether and to what extent the exposure to carcinogenic and non-carcinogenic compounds of importance and the consequent health risk is affected by consumers’ preference for spirits produced from specific types of fruit, taking into account four scenarios of gender-specific consumption of spirits: (1) population average per capita (aged 15+), (2) regular drinkers only (aged 15+ minus abstainers), (3) version A—chronic heavy drinkers (share of recorded and unrecorded alcohol) and (4) version B—chronic heavy drinkers (only recorded or unrecorded spirits) (Supplementary Material, Table S2). 

**Figure 7:**
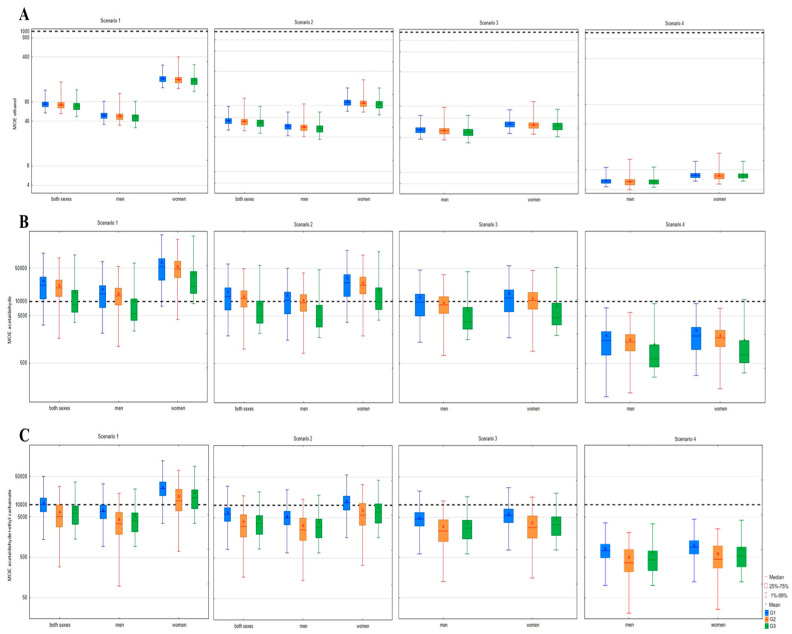
Margin of exposure (MOE) of carcinogenic volatiles in fruit spirits. (**A**)—ethanol, (**B**)—acetaldehyde, (**C**)—acetaldehyde + ethyl carbamate; ------------ cut-off point; G1—pome fruits; G2—stone fruits; G3—grape pomace.

The calculated MOE values for carcinogenic compounds are presented in Figure 7. The lowest MOE values and the highest health risk were recorded in the case of ethanol. Regardless of the gender and type of fruit from which the fruit spirits were made, ethanol already in scenario 1, which represents the average per capita consumption (aged 15+) of fruit spirits, showed MOE values far below the permitted limit of 1000. Even the highest mean MOE, recorded in the G1 group for women, was 187 (Figure 7A). At the highest risk were chronic male consumers, which is in accordance with the findings from Kokkinakis, et al. [37] (mean value of 4.74). Regarding acetaldehyde, already in scenario 1, a high proportion of the samples in all observed fruit classes was responsible for MOE values under the defined limit of 10,000 for men, women and both sexes, whereas the G3 group—grape pomace spirits—exerted the highest risk (Figure 7B). Almost 73% of spirit samples were hazardous for men in the G3 group, while in the G1 and G2 groups, these percentages were lower, 32% and 38%, respectively. The explanation for the higher quantity of acetaldehyde and consequent higher risk for grape pomace spirits could be found in the production process of a portion of our samples. Namely, grape pomace spirit production can be carried out separately from wine production, meaning that grape pomace is stored in containers causing the formation of larger quantities of acetaldehyde [38]. As published in the paper of Kusonić, Bijelić, Kladar, Torović and Srđenović Čonić [27], the highest range of MOE values for ethyl carbamate was observed in the G2 group and across the consumption scenarios. A larger share of stone fruit spirits demonstrated toxic potential compared to the remaining two groups of fruit spirits, which is in accordance with the statistically higher ethyl carbamate content in this group of products. To evaluate the cumulative risk of acetaldehyde and ethyl carbamate, considering their similar mechanism of action, the combined MOE (MOEc) was calculated (Figure 7C). All three groups of spirits showed an increased risk to human health starting from the average consumption scenario, whereby the most dominant hazardous spirits were those produced from stone fruits. For example, 95% of samples from this group presented a risk for men’s health versus 86% from G3 and 80% from group G1. Depending on the fruit class, the combined risk was more than two-, three- or even fourfold higher than the individual risk for acetaldehyde and ethyl carbamate, respectively, even in the average consumption scenario. Specifically, 95% of all samples in the G2 group were hazardous for men, while regarding only acetaldehyde or ethyl carbamate, these percentages were lower, 38% and 59%, respectively. More pronounced differences in combined and individual risk were noticed in pome fruit spirits, since 80% of samples were hazardous for men versus 32% and 29% when only acetaldehyde or ethyl carbamate were taken into account, respectively. 

**Figure 8:**
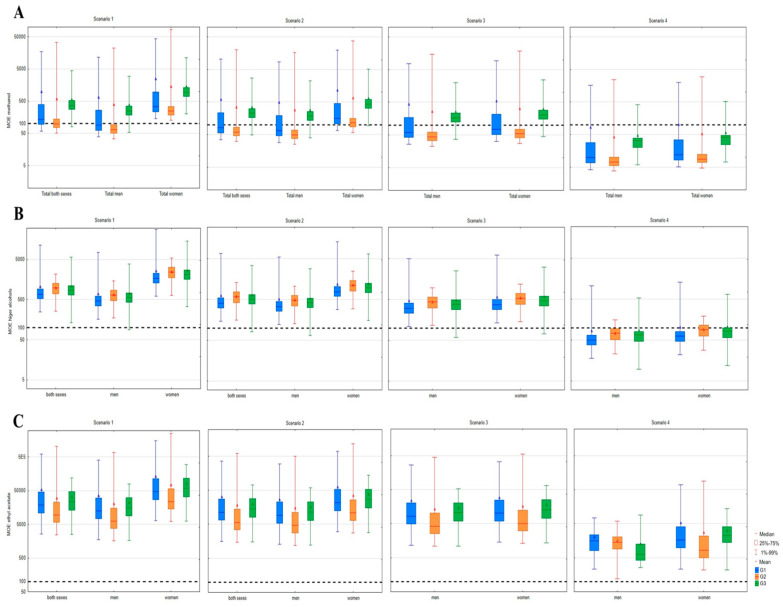
Margin of exposure (MOE) of non-carcinogenic volatiles in fruit spirits. (**A**)—methanol, (**B**)—higher alcohols, (**C**)—ethyl acetate; ------------ cut-off point;; G1—pome fruits; G2—stone fruits; G3—grape pomace.

Regarding non-carcinogenic compounds, the greatest risk to human health was posed by methanol (Figure 8A) in all fruit classes. Already in the first consumption scenario in the male population, 83% of spirits produced from stone fruits and almost 55% of pome fruit spirits posed a risk, compared to only 13% of grape pomace spirits. Namely, the lowest MOEs were observed for spirits from the G2 group, where the lower quartile MOE in the average consumption scenario was below 100 (MOE 70), while in the G1 group, this value was slightly below the cut-off point (MOE 94), and in the G3 group, it was higher (MOE 251). Concerning the sum of higher alcohols (Figure 8B), one of the samples from the G3 group, which had an almost twofold higher concentration of higher alcohols than other measured samples, posed a risk in men in the first three scenarios, while in the G1 and G2 groups of spirits, the risk was not observed. A high percentage of spirits exerted a risk for both men and women only in the case of the version B scenario (chronic heavy drinkers). Almost all sample spirits from group G1 posed a risk for men (96%) and women (93%), while these percentages were lower for the G2 group at 83 and 60% and the G3 group at 81 and 77% for men and women, respectively. This is in accordance with the significantly higher content of individual higher alcohols measured in pome fruit spirit samples. Only when the mean MOE was taken into consideration were exclusively chronic heavy drinkers of both genders at risk, regardless of fruit class. The possibility of harmful effects of ethyl acetate in any of the consumption scenarios was not under the influence of the fruit class—both men and women were not at risk (Figure 8C).

Considering all hazards assessed in this study, consumers of stone fruit spirits are at the highest risk. In our sample collection, more than 50% of all stone fruit spirits were from plum, and in Serbia, slivovitz is by far the most favorite fruit spirit among consumers. It has even been added to the UNESCO Representative List of the Intangible Cultural Heritage of Humanity. 

### 3.4. Compositional Analysis of Aromatic Compounds in Spirits in Relation to Fruit Source 

The quality of fruit distillates is influenced by various factors, with the type and quality of fruit raw material as two of the main ones. The comparison of the aromatic compounds of the fruit spirits in this study indicates a significant effect of the fruit source used for spirit distillation. Table 1 presents the abundance of all substances quantified in amounts ≥5% in the spirits of the selected fruits. The aromatic volatiles were made up of alcohols, esters, carbonyl compounds, terpenes and other compounds, with the alcohols being the most abundant in pome and stone fruits and esters in grape pomace spirits, representing even 50% of all aromatic volatiles. The occurrence of total alcohols was most dominant in the G1 group, in apple spirits especially, and the main alcohol contributors were 1-hexanol and 1-octanol. As for the esters, the most abundant components were benzoic acid ethyl ester and 2-methyl ethyl ester of butanoic acid, the occurrence of which was most pronounced in stone fruit spirits. Benzaldehyde, as a carbonyl compound, occurred in spirits produced from all fruit sources investigated. Terpenes occurred in apricot spirits in some significant numbers, while other components had less or more pronounced occurrence in spirits of all fruit sources. Differences in occurrence and amount of aromatic volatiles contribute to spirit flavor, but discussion of this is out of the scope of our study, i.e., semiquantitative analysis of aromatic compounds in fruit spirits was conducted to identify quality markers for differentiation of specific fruits used for spirit production.

### 3.5. Quality Markers for Differentiation of Specific Fruits Used for Spirit Production

**Figure 9:**
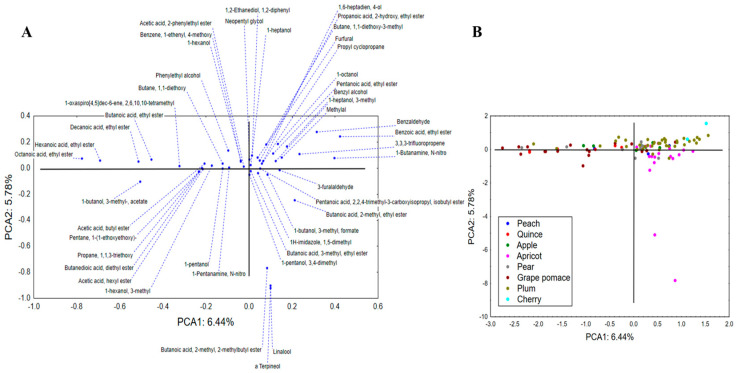
Principal component analysis—chemical profiling of aromatic compounds: principal components loadings (**A**) and positioning of analyzed spirit samples in the space defined by PCA1 and PCA2 (**B**).

High-quality distillates are products of tremendous branding potential and high economic value, and trade generates significant revenues for their producers. As such, they are often adulterated, copied or subject of other types of manipulation. The previously stated methods include blending with cheaper raw materials of lower quality, the addition of sugar during fermentation to obtain a higher yield of spirit, the addition of ethanol produced from cheaper raw materials (beet sugar, maize, cane sugar, grain, potato) or even the addition of synthetic alcohol [23,39]. Advanced analytical quality control could unveil such adulterations of fruit spirits and thereby contribute to the prevention of producer misconduct. The results of the semiquantitative analysis of aromatic compounds in collected spirits were processed by PCA, whereas only the compounds that were present in any of the samples in amounts ≥5% were used as input variables. Moreover, for aromatic profiling of samples, the type of fruit used as the starting raw material was set as a grouping variable. The PCA showed that the first two principal components describe a modest amount of initial variability, around 12% (Figure 9). The size of the variability in terms of PCA1 mostly correlated to the abundance of ethyl esters of hexanoic, octanoic, benzoic and decanoic acids, while the shape of the variability correlated to the amounts of α-terpineol, linalool and 2-methyl, 2-methylbutyl ester of butanoic acid. Positioning of the samples in the space defined by PCA1 and PCA2 (Figure 9) indicated a somewhat separative grouping in terms of PCA1. Namely, the positive space of PCA1 was reserved mostly for apricot, plum, cherry and pear spirits since they display a higher abundance of benzoic acid ethyl ester, benzaldehyde, benzyl alcohol and octanol. On the other hand, the samples of grape pomace and quince could be noticed in the negative part of PCA1 since they contain higher amounts of ethyl esters of butanoic, hexanoic, octanoic and decanoic acids.

**Figure 10:**
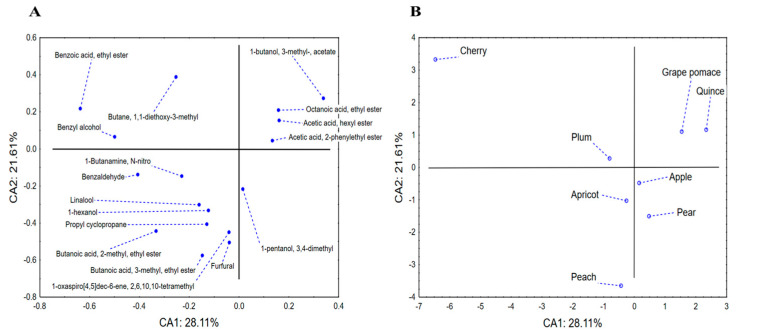
Canonical discriminant analysis—chemical profiling of aromatic compounds: loadings of the first two canonical roots (**A**) and positioning of analyzed spirit samples’ centroids in the space formed by the first two canonical axes (**B**).

**Figure 11:**
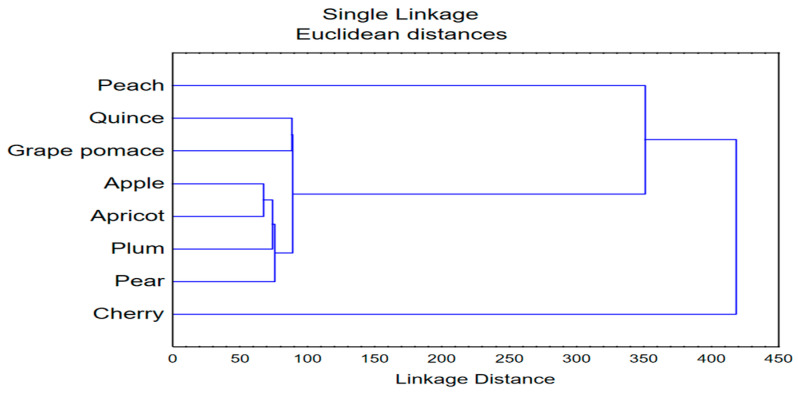
Cluster analysis dendrogram; input data—squared Mahalanobis distances obtained in CDA applied to the results of chemical profiling of aromatic compounds in spirits.

Furthermore, the same dataset (volatile substances) was analyzed by application of CDA. The proposed discriminant function indicated a model containing 17 variables of interest, whereas the first two roots (canonical discriminant axes, CAs) describe around 50% of samples’ discriminations (Figure 10). The size of recorded discriminations in terms of CA1 mostly correlated to the abundance of benzoic acid ethyl ester, benzyl alcohol and benzaldehyde, as well as 1-butanol-3-methyl acetate. On the other hand, the shape of discriminations (in terms of CA2) was highly affected by the amounts of 3-methyl-ethyl ester of butanoic acid, 2-methyl-ethyl ester of butanoic acid, furfural and 1,1-dietoxy-3-methylbutane. The position of analyzed samples’ centroids in the space formed by the first two canonical axes indicates the separation of cherry samples in the negative part of CA1 as a consequence of the high abundance of benzoic acid ethyl ester. On the other hand, quince and grape brandies are located in the positive part of CA1 since they contain 1-butanol-3-methyl acetate and lower amounts of benzoic and butanoic acid esters. Although being positioned in different parts of CA1, apple, pear, apricot and plum spirits display close grouping. The cluster analysis performed on squared Mahalanobis distances (Figure 11) clearly depicts previously stated discriminations within analyzed fruit spirits.

The application of multivariate statistics enabled the identification of several compounds which could assist in the authentication of fruit spirits and the distinguishing of spirits of different fruit origin. Namely, volatile compounds such as benzoic acid ethyl ester, benzyl alcohol, benzaldehyde, 3-methyl-ethyl ester of butanoic acid, 2-methyl-ethyl ester of butanoic acid and furfural were identified as the most notable “markers” for the reliable classification of the samples.

Results obtained for potentially harmful substances in spirits from different fruit classes indicate the need to take measures aimed at reducing the content of potentially toxic compounds, especially those that in spirits of a certain raw material origin cause the greatest risk to human health, such as ethyl carbamate, which showed a higher concentration and potential risk in stone fruit spirits; methanol in stone and pome fruit spirits; and acetaldehyde in grape pomace spirits. The volatile profile of the spirits indicates the fruit source, the technological conditions and the distillation technique used. For example, in the production process, the temperature applied in the kettle can induce chemical reactions among compounds, forming other substances and enhancing the complexity of the final distillate. It can influence the quality of the final product since there are unpleasant or even toxic compounds that could be present such as ethyl carbamate [40]. Very often, in traditional (homemade) spirit production, the fruit material is spontaneously fermented in wooden tanks, without any control, causing higher methanol content, especially with fruits with high pectin content such as pome or stone fruits [33]. Moreover, traditional plum spirit manufacture uses a simple and often single-pot still without adequate distillation cuts. Consequently, a brandy with a high content of high total acids, aldehydes, total esters and ethyl carbamate can be produced [41,42]. Acetaldehyde content depends on the strains of yeasts, the fermentation process and the manner of distillation cut. To reduce its content, distillation should be performed promptly after fermentation is finished. Thus, potentially harmful volatiles could be decreased to some extent with optimized production. Defining compounds characteristic of spirits obtained from different fruits could improve the quality control of this type of product since its production often represents an area susceptible to manipulation regarding both raw materials and the production process due to the possibility of great economic profit. This is of particular importance especially if considering the overall availability of fruit spirits from unregistered production that are not subjected to safety and quality control. Additionally, the production processes are not standardized and rather rely on producers’ knowledge passed through generations.

## 4. Conclusions

Chemometric exploration of the distribution patterns of potentially harmful volatile compounds and toxic metal(loid)s in fruit spirits relating to distinct fruit classes most commonly used in spirit production, i.e., pome fruits, stone fruits and grape pomace, highlighted the potential of several volatiles as candidates for differentiation markers while dismissing toxic metal(loid)s. Namely, pome fruit and grape pomace spirits were mostly characterized by a lower abundance of n-propanol, methanol, ethyl acetate and even acetaldehyde and stone fruit spirits by lower amounts of isoamyl alcohol and isobutanol. Additionally, chemometric analysis of the composition of aromatics in fruit spirits identified additional potential markers characteristic of certain fruit classes, such as ethyl ester of benzoic acid, benzyl alcohol, benzaldehyde, 3-methyl-ethyl ester of butanoic acid, 2-methyl-ethyl ester of butanoic acid and furfural.

Apart from being able to tell the difference between the fruit sources of spirits, this study focused on exploring differences in the risk potential of investigated spirits, considering that some chemicals known to be detected in spirits are potent health hazards. On one hand, it would be rather contradicting to propose compounds posing serious health risks as differentiation markers, because safety must be the overriding factor. On the other hand, it is of great interest to perceive to what extent health risks are affected by consumers’ preference for spirits of distinct fruit types. Regarding the presented issues, the study findings showed that consumers of stone fruit spirits are potentially at higher risk than those consuming pome fruit or grape pomace spirits.

Altogether, while differentiation of fruit spirits based on markers revealed by chemometric analysis shall contribute to the prevention of economically motivated adulteration, differentiation based on the risk posed by their chemical composition should facilitate informed choices—as previously mentioned, a substantial number of people are willing to accept the risk posed by ethanol, mostly not taking it seriously because ethanol is a well-known and still widely accepted stimulant, but being aware of a multitude of other harmful compounds potentially present in a shot of their favorite spirit can break the myth that a shot of rakija in the morning heals everything. Only then might the choice “To drink, or not to drink alcohol”? replace the dilemma “Plum or quince (or …) rakija for me”? Before such a transition, it is not to be expected that people will give up alcohol.

## Figures and Tables

**Table 1:** The abundance of all aromatic substances quantified in amounts ≥5% in spirits of selected fruits.

	Abundance (%)							
	G1—Pome Fruit			G2—Stone Fruit				G3—Grape Pomace
	Apple	Pear	Quince	Plum	Peach	Apricot	Cherry	Grape
**COMPOUND**	N = 7	N = 10	N = 7	N = 38	N = 3	N = 19	N = 2	n = 20
**TOTAL ALCOHOLS**								
1-pentanol	14	20	14	5	0	11	0	15
1-hexanol	86	40	29	13	0	32	0	30
1-octanol	57	30	43	32	33	32	50	35
Phenylethyl alcohol	29	20	43	11	0	16	0	25
1-hexanol, 3-methyl	14	0	14	0	0	0	0	0
1-heptanol	14	10	0	0	0	5	0	0
Neopentyl glycol	0	0	0	0	0	0	0	0
1-pentanol, 3,4-dimethyl	0	10	0	0	0	0	0	0
1,6-heptadien, 4-ol	0	0	0	3	0	0	0	0
Benzyl alcohol	0	0	0	3	0	0	50	0
1,2-Ethanediol, 1,2-diphenyl	0	0	0	5	0	0	0	0
1-heptanol, 3-methyl	0	0	0	3	0	0	0	0
**TOTAL ESTERS**								
Butanoic acid, ethyl ester	29	0	43	13	0	16	0	30
1-butanol, 3-methyl-, acetate	29	50	86	58	67	68	0	70
Hexanoic acid, ethyl ester	57	70	71	50	0	16	50	70
Acetic acid, hexyl ester	0	0	14	0	0	0	0	0
Octanoic acid, ethyl ester	57	70	86	45	33	37	0	70
Decanoic acid, ethyl ester	14	10	29	11	33	0	0	25
Propanoic acid, 2-hydroxy, ethyl ester	0	20	0	3	0	5	0	0
Benzoic acid, ethyl ester	43	10	0	84	67	32	100	5
Acetic acid, 2-phenylethyl ester	0	0	14	0	0	0	0	0
Butanoic acid, 2-methyl, ethyl ester	29	50	0	37	67	84	0	10
Butanedioic acid, diethyl ester	14	20	0	11	0	0	0	10
Pentanoic acid, ethyl ester	0	0	0	16	0	5	0	0
Butanoic acid, 3-methyl, ethyl ester	14	10	0	13	67	5	0	5
1-butanol, 3-methyl, formate	0	0	0	3	0	5	0	0
Butanoic acid, 2-methyl, 2-methylbutyl ester	0	0	0	0	0	5	0	0
Pentanoic acid, 2,2,4-trimethyl-3- carboxyisopropyl, isobutyl ester	0	0	0	3	0	0	0	0
Acetic acid, butyl ester	0	0	0	3	0	0	0	0
**TOTAL CARBONYL COMPOUNDS**								
Benzaldehyde	100	60	43	82	33	26	100	10
Furfural	29	30	14	58	67	37	0	5
3,3,3-trifluoropropene	0	0	14	11	0	5	0	0
Propyl cyclopropane	0	0	14	3	33	5	0	0
Butane, 1,1-diethoxy-3-methyl	0	0	0	3	0	0	50	10
3-furalaldehyde	0	0	0	5	0	11	0	0
Benzene, 1-ethenyl, 4-methoxy	0	0	0	3	0	0	0	0
Propane, 1,1,3-triethoxy	0	0	0	0	0	0	0	5
Butane, 1,1-diethoxy	0	0	0	0	0	0	0	15
Pentane, 1-(1-ethoxyethoxy)-	0	0	0	0	0	0	0	5
**TOTAL TERPENES**								
Methylal	0	0	0	3	0	0	0	0
α-Terpineol	0	0	0	0	0	37	0	0
Linalool	0	0	0	0	0	42	0	10
**OTHER COMPOUNDS**								
1-Butanamine, N-nitro	0	20	0	47	67	53	50	10
1-Pentanamine, N-nitro	43	20	14	21	33	11	0	15
1H-imidazole, 1,5-dimethyl	14	10	0	5	33	11	0	5
1-oxaspiro[4,5]dec-6-ene, 2,6,10,10-tetramethyl	0	20	29	0	0	0	0	0

## Data Availability

The data for this work were obtained from previously published research and are available (https://doi.org/10.1016/j.arabjc.2022.103981, https://doi.org/10.1080/19393210.2023.2262956, https://doi.org/10.1016/j.jfca.2022.104807 (accessed on 28 September 2024)) with the permission of the authors (Branislava Srdjenović Čonić branislava.srdjenovic-conic@mf.uns.ac.rs, Dejan Kusonić dejan.kusonic@mf.uns.ac.rs, Ljilja Torović ljilja.torovic@mf.uns.ac.rs).

## Supplementary Materials

The following supporting information can be downloaded at: https://www.mdpi.com/article/10.3390/toxics12100720/s1, Table S1: GC-MS analysis parameters; Table S2: Volumes of per capita consumption of recorded and unrecorded spirits in the Republic of Serbia (L of pure alcohol per year). Reference [43] is cited in the supplementary materials.
