# Supplementary material for: A Chemometric Exploration of Potential Chemical Markers and an Assessment of Associated Risks in Relation to the Botanical Source of Fruit Spirits
Source: Toxics. 2024 Oct 2;12(10):720. doi: 10.3390/toxics12100720 (PMC11511030; doi:10.3390/toxics12100720)
Supplement: Supplementary file 1 [file toxics-12-00720-s001.zip › toxics-3222162-supplementary.pdf]

toxics

MDPI

# A Chemometric Exploration of Potential Chemical Markers and an Assessment of Associated Risks in Relation to the Botanical Source of Fruit Spirits

**Branislava Srdjenović Čonić [1,2], Nebojša Kladar [1,2], Dejan Kusonić [1], Katarina Bijelić [1,2,*] and Ljilja Torović [1,2]**

**Table S1.** GC-MS analysis parameters.

| Gas Chromatograph (GC) | |
|---|---|
| **PARAMETERS** | **CONDITIONS** |
| Inlet | 220°C, splitless |
| Helium carrier gas flow rate | 1 mL/min |
| MSD transfer line temperature | 240°C |
| Oven starting temperature | 40°C; hold time: 5 min |
| Ramp 1 | 180°C, 3°C/min; hold time: 0 min |
| Ramp 2 | 280°C, 40°C/min; hold time: 5 min |
| **Mass detector (MD)** | |
| Acquisition mode | Scan |
| Solvent delay | 7.5 min |
| Scan parameters | m/z = 50 - 550 |
| Source temperature | 230 (max 250°C) |
| Quadrupole temperature | 150 (max 200°C) |
| Column | HP5-MS, 30m x 0.25mm x 0.25 µm |

**Table S2.** Volumes of per capita consumption of recorded and unrecorded spirits in the Republic of Serbia (L of pure alcohol per year).

| | **Scenario 1** | | **Scenario 2** | | **Scenario 3** | | **Scenario 4** | |
|---|---|---|---|---|---|---|---|---|
| | **average consumption** | | **regular drinkers only** | | **chronic heavy drinkers (A)** | | **chronic heavy drinkers (B)** | |
| **Spirits** | recorded | unrecorded | recorded | unrecorded | recorded | unrecorded | recorded | unrecorded |
| | *litres of pure alcohol per capita yearly* | | | | | | | |
| **Men** | 3.89 | 3.00 | 5.28 | 4.07 | 5.84 | 4.50 | 27.76 | 27.76 |
| **Women** | 0.86 | 0.66 | 1.85 | 1.43 | 3.90 | 3.00 | 18.50 | 18.50 |
| **Both sexes** | 2.34 | 1.80 | 3.89 | 3.00 | | | | |

All input data necessary for the calculation of alcohol consumption volumes, presented in Table 1, were taken from the WHO Global Status Report on Alcohol and Health (WHO, 2018).

WHO. (2018). Global status report on alcohol and health 2018. Retrieved from https://apps.who.int/iris/handle/10665/274603
